# IgE autoantibodies and autoreactive T cells and their role in children and adults with atopic dermatitis

**DOI:** 10.1186/s13601-020-00338-7

**Published:** 2020-08-03

**Authors:** Fariza Mishaal Saiema Badloe, Shauni De Vriese, Katarina Coolens, Carsten B. Schmidt-Weber, Johannes Ring, Jan Gutermuth, Inge Kortekaas Krohn

**Affiliations:** 1 Department of Dermatology, SKIN Research Group, Vrije Universiteit Brussel (VUB), Universitair Ziekenhuis Brussel (UZ Brussel), Laarbeeklaan 103, Building D, Room D148, 1090 Brussels, Belgium; 2 Center of Allergy and Environment (ZAUM), Technical University and Helmholtz Center Munich, Munich, Germany; 3 Member of the German Center of Lung Research (DZL) and the Helmholtz Initiative for Inflammation and Immunology (I&I), Munich, Germany

**Keywords:** Atopic dermatitis, Autoallergens, Autoreactive T cells, Autoreactivity, IgE autoantibodies

## Abstract

The pathophysiology of atopic dermatitis (AD) is highly complex and understanding of disease endotypes may improve disease management. Immunoglobulins E (IgE) against human skin epitopes (IgE autoantibodies) are thought to play a role in disease progression and prolongation. These antibodies have been described in patients with severe and chronic AD, suggesting a progression from allergic inflammation to severe autoimmune processes against the skin. This review provides a summary of the current knowledge and gaps on IgE autoreactivity and self-reactive T cells in children and adults with AD based on a systematic search. Currently, the clinical relevance and the pathomechanism of IgE autoantibodies in AD needs to be further investigated. Additionally, it is unknown whether the presence of IgE autoantibodies in patients with AD is an epiphenomenon or a disease endotype. However, increased knowledge on the clinical relevance and the pathophysiologic role of IgE autoantibodies and self-reactive T cells in AD can have consequences for diagnosis and treatment. Responses to the current available treatments can be used for better understanding of the pathways and may shed new lights on the treatment options for patients with AD and autoreactivity against skin epitopes. To conclude, IgE autoantibodies and self-reactive T cells can contribute to the pathophysiology of AD based on the body of evidence in literature. However, many questions remain open. Future studies on autoreactivity in AD should especially focus on the clinical relevance, the contribution to the disease progression and chronicity on cellular level, the onset and therapeutic strategies.

## Background

Atopic dermatitis (AD) is an itchy, chronic relapsing skin condition affecting up to 25% of children and 2–8% of adults [1, 2]. First manifestations of AD usually appear in early childhood [3]. Allergic sensitizations in early childhood can act as eliciting or aggravating factors (atopic march) [4], which may continue in adulthood [5, 6] and exert a major disease burden, impaired quality of life and individual suffering. AD is an environmentally induced and IgE-mediated disease in which the allergic inflammation is causing a disturbed skin barrier, and can be aggravated by colonization with *Staphylococcus aureus* [7], *Malassezia* species [8–10] and pollutants [11].

The association between atopy and autoimmune diseases has gained interest in the last decades, likely because the incidences of both allergic- (AD, asthma, and rhinoconjunctivitis, allergic rhinitis) and autoimmune diseases (e.g. psoriasis, multiple sclerosis) are rising worldwide [12]. Patients with AD can be at higher risk for the development of co-morbid autoimmune diseases [13–15]. In addition, a combined allergic-autoimmune-driven response has been described in patients with moderate/severe AD [15–22]. However, it is still unclear whether IgE autoreactivity could be an endotype of AD or an epiphenomenon [21, 23]. Although several studies associate the presence of IgE autoreactivity with AD, the clinical relevance needs yet to be further investigated. Currently, the prevalence of autoreactive antibodies has mainly been investigated in adult patients with different disease backgrounds and age-matched healthy controls, while the pediatric profile is not well characterized. Development of autoreactive antibodies may already start in early childhood [20], likely due to the lack of immune stimulation. However, increased understanding of autoallergy in children may be of great importance with direct consequences for diagnosis and therapy.

**Fig. 1:**
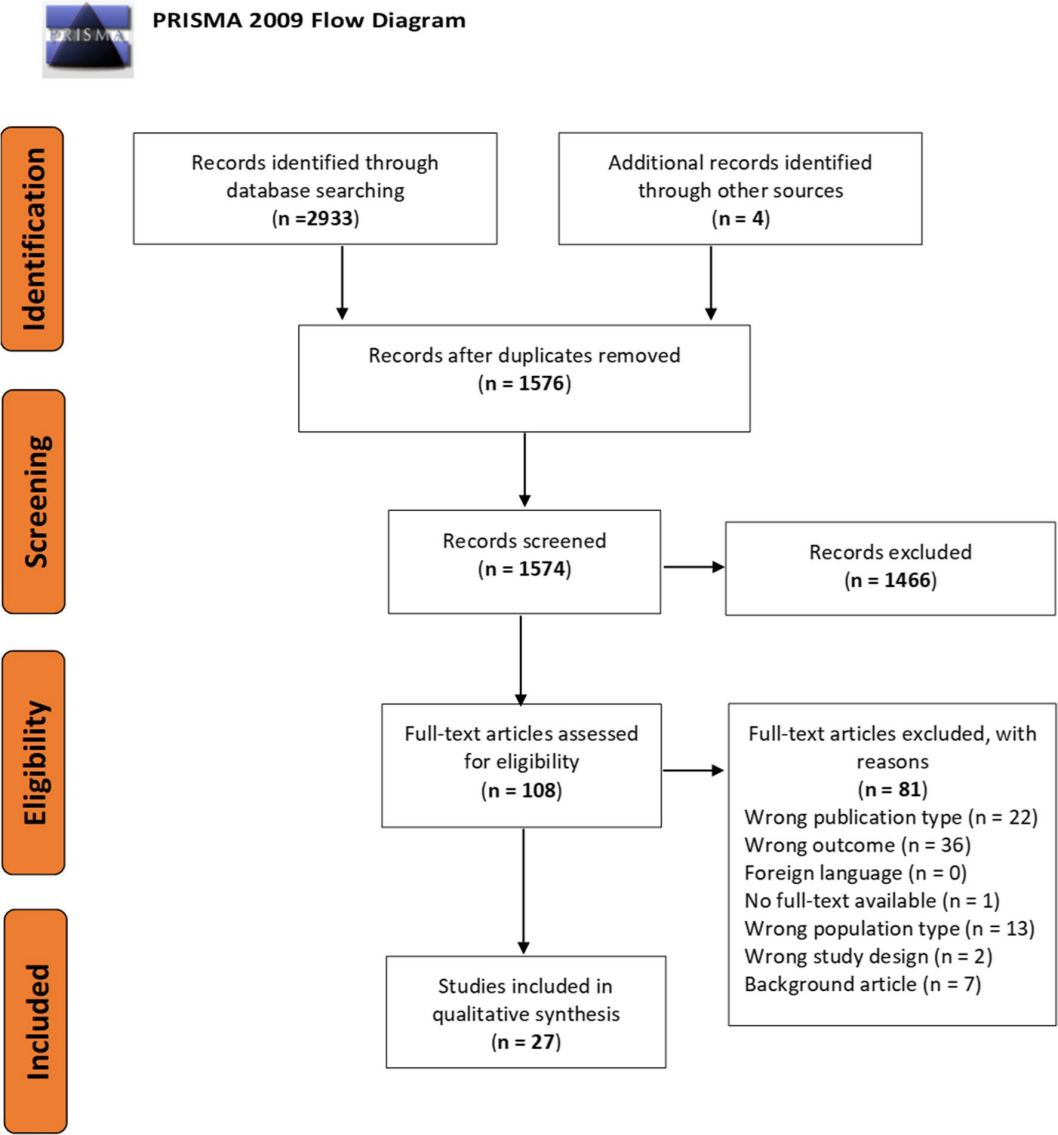
Flow diagram of the systematic search

The aim of this review is to summarize evidence on IgE autoreactivity in AD and the possible cellular pathways contributing to disease chronicity and severity. Additionally, we aim at comparing autoreactive profiles in children, adolescents and adults with AD to provide an overview of current knowledge and gaps. A systematic search was performed in PubMed using the following search strategy: Atopic dermatitis, atopic eczema, autoreactive, auto-IgE, autoantigen, autoallergy, autoimmunity, autoantibodies, autoreactive T cells (Additional file 1: Table S1). All available original studies on the association of immunoglobulin E (IgE) autoantibodies and T lymphocyte (T cell) autoreactivity in patients with AD were included. Studies in languages other than English, French, Dutch and German were excluded. The last update was on October 3, 2019. Eligible studies were screened by two independent reviewers (FB, KC) on title and abstract. Screening of full-text and data-extraction was performed (FB and SDV) and disagreements were resolved by discussion with a third reviewer (IKK). The PRISMA flow diagram [24] was used to depict the flow of the selection process (Fig. 1). In total, 27 original articles were included of which 18 on IgE autoantibodies, 7 on autoreactive T cells, 1 on IgG autoantibodies and 1 on sweat antigen (Fig. 1). An overview of the original articles on IgE autoreactivity in patients with AD can be found in Additional file 1: Table S2.

## The association of atopic dermatitis and autoimmunity

Atopy has been associated with classical autoimmune disorders and AD may co-occur with autoimmune diseases (psoriasis, rheumatoid arthritis, multiple sclerosis and type I diabetes mellitus). In a matched case–control study, including 8589 children with diagnosed AD before the age of 5 and 85,890 controls, the association between parental autoimmune disease and the development of AD in their offspring were studied [25]. The presence of maternal (not paternal), dermatologic or digestive autoimmune disease was associated with an increased risk for development of AD in their offspring. A follow-up study on a previous cohort consisting of 241 children with AD based on a questionnaire with a response of 145 adolescents (16–23 years), found an increased incidence of autoimmune disorders compared to non-atopic controls (9% vs. 1%) [26]. This suggested that infantile AD may increase the risk of autoimmune disorders in adulthood. A study on 8112 adult patients with AD and age and gender matched controls demonstrated an association of AD with 11 of 22 selected autoimmune diseases. Additionally, AD was linked with having multiple autoimmune comorbidities, and smoking increased the risk of autoimmune comorbidities compared to nonsmokers [15]. However, a study including 14,849 subjects from five cohorts evaluated the relation between specific-IgE positivity against airborne allergens and autoimmunity, but no associations between atopic predisposition and development of autoimmune disorders were found [27]. Contrarily, IgE-mediated diseases may reduce the risk of developing autoimmunity. Active T helper 1 (Th1)-inflammation may suppress development of atopy, and atopy may suppress the severity of autoimmunity [28, 29]. As a result of contradictory findings and varying study protocols, the underlying mechanism of AD and autoimmune disease comorbidities is still unclear.

## IgE autoreactivity in atopic dermatitis

Patients with AD often display elevated levels of total serum IgE without clinically relevant allergy for the most common allergens. Autoreactive IgE antibodies may elicit an allergic-autoimmune process and contribute to perpetuation of inflammation. This can be a reason for the chronic relapsing course of AD. Studies on the presence of a combined allergy-autoimmune process in patients with AD are summarized here.

### Prevalence of IgE autoreactivity in AD

The first evidence of the presence of IgE autoreactivity to human proteins in AD was described 25 years ago [16–19]. IgE autoantibodies were found in sera from patients with AD or have been determined via a positive response to autoantigens in vitro. The prevalence of autoreactivity in patients with AD was previously summarized and ranges from 23 to 91% based on 14 studies involving 2644 individuals [21]. Comparable results were obtained in the present review (Table 1, Additional file 1: Table S2). Most likely the variation in the prevalence of autoreactivity can be explained by different detection methods, cohort composition and the origin of the tested autoantigens. Autoreactive antibodies of IgG [30, 31] and IgM [32] have also been detected in patients with AD. In this review we only focus on IgE autoreactive antibodies.

**Table 1:** Prevalence of autoreactivity in patients with atopic dermatitis categorized by age

Patients with atopic dermatitis	Severity of AD/population	Prevalence of IgE autoreactivity in AD	Prevalence of IgE autoreactivity in control subjects	References
Children 0–1 year	Mild–severe	20.0% (1/5)	/	Natter et al. [19]
	Moderate–severe	15.0% (25/60)	/	Mothes et al. [20]
Children 2–13 years	Mild–severe	16.7% (1/6)	/	Natter et al. [19]
	Moderate–severe	80.0% (16/20)	/	Mothes et al. [20]
Adolescents 14–23 years	Mild–severe	63.6% (7/11)	/	Natter et al. [19]
	EASI: 10–39.4	50.0% (2/4)	/	Mitterman et al. [38]
Adults ≥ 24 years	/	60.0% (12/20)	0.0% (0/2)	Valenta et al. [17]
	Mild–severe	44.8% (13/29)	/	Natter et al. [19]
	/	62.5% (10/16)	0% (0/1)	Ochs et al. [57]
	/	2.5% (1/40) SART2_161_	4.9% (2/41) SART2_161_	Kawamoto et al. [30]
	/	37.5% (15/40) SART_109_	29.3% (12/41) SART_109_	
	/	15% (6/40) SART_315_	17.1% (7/41) SART_315_	
	/	27.5% (11/40) CypB_84_	29.3% (12/41) CypB_84_	
	/	17.5% (7/40) CypB_91_	31.7% (13/41) CypB_91_	
	/	10% (4/40) ART4_75_	12.2% (5/41) ART4_75_	Kinaciyan et al. [53]
	Mild	100.0% (1/1)	/	Kortekangas-Savolainen [33]
	/	37.0% (10/27)	/	Aichberger et al. [37]
	/	91.7% (11/12)	0.0% (0/6)	Mothes et al. [20]
	/	23.0% (40/174)	/	Schmid-Grendelmeier et al. [34]
	EASI: 0.6–39.4	42.0% (29/69)	/	Tanaka et al. [55]
	Mild–severe	77.0% (47/61)	8.7% (4/46)	Mitterman et al. [38]
	EASI: 4.4–59.8	85.7% (6/7)	0.0% (0/12)	Altrichter et al. [35]
	EASI: 0.6–39.4	28.0% (54/192)	/	Zeller et al. [36]
	SCORAD: 21.93–71.36	71.8% (51/71)	0.0% (0/36)	Watanabe et al. [31]
	/	14.8% (9/61)	/	Aichberger et al. [37]
	/	91.7% (11/12)	0.0% (0/6)	Lucae et al. [91]
	Moderate–severe	100% (4/4)	/	Roesner et al. [106]
	/	91.7% (11/12)	/	

Percentage of IgE autoreactivity in atopic dermatitis with the severity of the disease compared with healthy subjects, subdivided into children 0–1 year, 2–13 years, adolescents and adults

The prevalence of IgE autoreactivity has mainly been studied in adults with AD [17, 33–37]. Only a few studies included children with AD [19, 20] or adolescents [19, 38], while AD usually starts in childhood and the percentage of children affected by AD is around 7 times higher than in adults. Due to insufficient numbers of studies on IgE autoreactivity both in children with AD and in healthy age-matched controls, the cause and thus, the start of the development of IgE autoantibodies is unknown.

It has been established that AD occurs more frequently in individuals with an Asian, African or Afro-American genetic background [39]. However, the association between ethnicity and the presence of IgE autoreactivity in AD has not been described so far.

### Autoreactivity may develop already in early childhood

Autoreactivity may develop in early infancy [20] and atopy in early childhood may increase the development of autoimmune diseases later in life [20, 26, 28]. In a cross-sectional study including 346 children with active AD and 117 controls antinuclear antibodies (ANA) have been found in both groups, which increased with age [40]. Although not significantly different, in children with AD the ANA antibodies had a tendency to appear earlier, suggesting that active AD in early infancy can lead to earlier development of systemic autoreactivity [40].

Only few studies investigated IgE autoantibodies in children, adolescents and adults with AD. One study found that 22/51 patients with AD were positive for autoreactive antibodies [19]. To evaluate the IgE autoreactivity based on age, we subdivided the study population (Table 1). Additionally, a retrospective analysis of serological parameters from both children and adults with AD gave insights in the onset of IgE autoreactivity [20]. Twenty-three percent of adults with AD showed IgE reactivity to human epithelial antigens compared to the control groups. In addition, 15% of children younger than 1 year of age showed IgE autoreactivity. Children at the age of 2–13 years with moderate to severe AD, 80% were positive for IgE autoantibodies. Repeated serum determinations within 1 year demonstrated increased IgE autoreactivity in children at 2–6 years with AD and multiple sensitizations concluding that IgE autoreactivity can develop in early infancy [20]. The prevalence of IgE autoantibodies in newborns, children, adolescents and adults with AD based on their age is presented in Table 1.

### Prevalence of IgE autoreactivity in other diseases

Often, AD is the starting point of the atopic march, in which patients with AD develop food allergy, allergic rhinitis, allergic rhinoconjunctivitis and allergic asthma later in life [41]. In case IgE autoantibodies start to develop in early childhood, it seems possible that these antibodies also play a role in co-morbid diseases of AD (and enhance the atopic march), as well as other inflammatory diseases. On the other hand, IgE autoantibodies have been identified in comparable frequency in children with or without AD (unpublished data Gutermuth and Schmidt-Weber). Thus, the pathophysiologic relevance of autoreactive IgE has not been investigated in-depth.

Other skin-related diseases have been associated with the presence of IgE autoreactive antibodies, such as chronic urticaria [42], IgE-mediated bullous pemphigoid [43]. Additionally, 65% of the patients with systemic lupus erythematosus produce IgE autoantibodies [44] and 83% in patients with active disease [45] as well as 50–60% of patients with rheumatoid arthritis [46]. Regarding other isotypes, in patients with rheumatoid arthritis, autoantibodies of subclasses IgG, IgM and IgA can be found in serum years before the disease onset and can predict disease development [47–50]. It was previously reported that IgE autoantibodies were neither present in healthy subjects nor in patients with allergic rhinoconjunctivitis, psoriasis or other inflammatory diseases based on results from 8 studies of 816 participants [21]. The prevalence of autoreactivity was not associated with age, gender, or disease duration, but rather linked to disease severity [21].

### The presence of IgE autoreactivity in atopic dermatitis has been linked to disease severity

Since the discovery of autoreactivity in AD, the presence of IgE autoantibodies has been associated with disease severity in adult patients [17, 19, 34, 35, 51–53] and in children with AD [20]. IgE autoantibodies were mainly found in patients with moderate to severe AD, while they were absent in healthy controls (Table 2). Besides, IgE autoantibody levels decreased after successful treatment [20]. Therefore, the presence of IgE autoantibodies is described as a pathogenic factor in AD and as an indicator for chronic tissue damage. However, some studies also tested healthy individuals positive for autoreactivity. Skin tests with intradermal autologous sweat injections were positive in 56/66 patients with AD (84.4%), and in 3/27 healthy subjects (11.1%) [54]. Another study showed sweat antigen-induced histamine release from basophils in 47/61 (74.6%) patients with AD and 4/46 (8.7%) healthy subjects [55]. In a study on the autoreactivity to eight different cytotoxic T-lymphocyte (CTL)-directed peptides, no differences in IgE autoreactivity to 7 out of 8 CTL-directed epitopes were observed in patients with AD compared to healthy controls, while IgG autoreactivity was missing in a significant fraction of healthy donors. However, the study lacks functional appraisal of this finding [30]. Eukaryotic translation initiation factor 6 (eIF6)-specific serum IgG could be detected both in AD and healthy subjects. In addition, patients with AD and IgE autoantibodies showed no differences in SCORAD index compared to patients with AD without IgE autoantibodies (*P *< .7625), but had higher levels of total serum IgE [36]. These contradictory findings in healthy subjects may depend on the age of the patients (unpublished data Gutermuth and Schmidt-Weber) or the targets that were tested (Table 2).

**Table 2:** Targets of IgE autoantibodies in atopic dermatitis

Human autoantigens	Synonym	Molecular weight (kDa)	Prevalence (% of autoreactive AD patients)	Healthy controls	Description	References
*Actin α*	ACTA2	42	15.5% (11/71)	0% (0/24)	Alpha smooth muscle actin	Zeller et al. [36]
*Autologous sweat*	MGL_1304	29	84.4% (56/66)* 77.0% (47/61)** 5–27% (children) 29–65% (adults)	11.1% (3/27)* 8.7% (4/46)**	Product from *Malassezia globosa*, sharing homologs with other *Malassezia* species, but no cross-antigenicity with human proteins	Hide et al. [54] Tanaka et al. [55] Glatz et al. [107]
*Cytotoxic T*-*lymphocyte (CTL)*-*directed epitopes:*	SART2_161_ SART3_109_ SART3_315_ ART4_75_ CypB_84_ CypB_91_ CypB	21	2.5% (1/40) 37.5% (15/40) 15% (6/40) 10% (4/40) 27.5% (11/40) 17.5% (7/40) 9.2% (6/71)	4.9% (2/41) 29.3% (12/41) 17.1% (7/41) 12.2% (5/41) 29.3% (12/41) 31.7% (13/41) 0% (0/24)	SART, ART4, CypB; highly expressed in proliferating cells SART; squamous cell carcinoma antigen. Tumor associated antigen recognized by CTL ART4; Adenocarcinoma antigens recognized by T cells 4 CypB; cyclophilin B, identified in all cell types in the endoplasmic reticulum, nucleus and in serum. Associated with progression of inflammatory diseases	Kawamoto et al. [30] Zeller et al. [36]
*Dense fine speckles (DFS70)/Lens epithelium derived growth factor (LEDGF)*		70	15% (9/61) 62.5% (10/16)	0% (0/20) 0% (0/1)	Survival factor, growth factor, HIV-transporter, nuclear antigen	Watanabe et al. [31] Ochs et al. [57]
*eIF6*		27	25.4% (19/71)	0% (0/24)	Eukaryotic translation initiation factor 6, nuclear and ribosomal protein	Zeller et al. [36]
*Epidermal keratinocytes*			37% (10/27)	0% (0/6)		Kortekangas-Savolainen et al. [33]
*Epidermis*			22% (12/54)	0% (0/138)		Altrichter et al. 35]
*Epithelial cell line A431*		25–97	43.1% (22/51) 60% (12/20) 91.7% (11/12) 17% (9/54) 72.7% (8/11) 23% (40/174)	0% (0/1) 0% (0/2) 0% (0/6) 0% (0/138) 0% (0/26)		Natter et al. [19] Valenta et al. [17] Aichberger et al. [37] Altrichter et al. [35] Mittermann et al. [38] Mothes et al. [20]
*HLA*-*DR*		24	8.7% (7/71)	0% (0/24)	Human leukocyte antigen, expressed on Langerhans cells, macrophages, keratinocytes and dendritic cells	Zeller et al. [36]
*Hom s 1*	SART-1	55–60			Cytoplasmatic and nuclear protein, SART1 almost identical sequence homology with Hom s1	Valenta et al. [18]
*Hom s 2*	α-NAC	23.2	27.3% (3/11)		Nascent polypeptide-associated complex, subunit α. Regulation of gene transcription	Mittermann et al. [38] Mossabeb et al. [108]
*Hom s 3*	BCL7B	20.1			Sequence identity with intracellular oncoprotein BCL7B. Positive regulator of apoptosis, maintenance of nuclear structure, stem cell differentiation	Natter et al. [19]
*Hom s 4*	MICU1, CALC	54	16.7% (2/12)	0% (0/6)	Basic protein which belongs to subfamily of calcium-binding proteins	Aichberger et al. [37]
*Hom s 5*	Cytokeratin 6A, K6A, KRT6D	56–70			Keratin type II cytoskeletal 6A. Structural constituent of cytoskeleton	Natter et al. [19]
*Human manganese superoxide dismutase (hMnSOD)*	Asp f6	22	42% (29/69) 14.8% (4/27) 17,6% (adults, 3/17)* 10% (children, 1/10)*	0% (0/5) 0% 0/27) 0% (0/17)* 0% (0/10)*	Nuclear-encoded enzyme, located in mitochondria. Cross reactivity with Mala s 11 and Hev b 10	Schmid-Grendelmeier et al. [34] Guarneri et al. [109] Vilhelmsson et al. [110] Andersson et al. [111]
*Human thioredoxins (hTrx)*		12	5% (3/61) 23.4% (16/71)	0% (0/20) 0% (0/24)	Small intracellular redox proteins involved in many important biological processes. 45% identity with Mala s13	Watanabe et al. [31] Zeller et al. [36] Schmid-Grendelmeieret al. [8] Limacher et al. [66]
*IgE, CH3 and CH4 domains*			Unknown (n = 19 AD)	Unknown (n = 13 HC)		Czech W et al. [112]
*Ribosomal protein P2*	RPLP2, Asp f8				Ribosomal phosphoprotein with a role in the elongation step of protein synthesis	Mayer et al. [113]
*RP1*	MAPRE2	39	29.0% (21/71)	0% (0/24)	Microtubule polymerization and stabilizing function	Zeller et al. [36]
*Tubulin α 1a*	TUBA1a	50	21.7% (14/71)	0% (0/24)	Formation of microtubules for cytoskeletal structure	Zeller et al. [36]

* Investigated using skin prick test, not specific auto-IgE

** Investigated using histamine release from basophils

When IgE autoantibodies are present in patients with severe disease, the level of autoantibodies may already increase before appearance of symptoms, as has been shown for IgG in patients with rheumatoid arthritis [47–50]. If so, the expansion of IgE autoantibodies may be useful as a marker or predictor for increased disease activity. However, prospective observational studies in children, adolescents and adults or birth cohorts are needed to investigate this. Alternatively, IgE autoreactivity might be an epiphenomenon which is elicited by a Th2-driven inflammation.

### Targets of IgE autoantibodies

The first targets of IgE autoantibodies (autoantigens) that have been identified are epitopes of human keratinocyte proteins with partly unknown function (Hom s 1–5) [17–19, 37]. In more recent years, a wide range of autoantigens have been described. The most relevant targets are summarized in Table 2 and were previously listed by others [21, 22, 56]. Patients with the highest total serum IgE values, showed the highest frequency of anti-keratinocyte IgE [33]. Total protein purifications from epidermal carcinoma cell line A431 (17–91.7%) and epidermal keratinocyte suspensions (37%) were used to evaluate the presence of IgE autoreactivity in adult patients with AD. As specific targets, human manganese superoxide dismutase (MnSOD, 37.2%), RP1 (29%), eukaryotic translation initiation factor 6 (eIF6, 25.4%), dense fine speckles (DFS, 24.7%), Hom s2 (27.3%), Hom s4 (16.7%) and human thioredoxins (hTrx, 14.4%) have shown the highest percentages of autoreactivity, while no autoreactivity in healthy individuals (0%) was found for these targets (Table 2). This suggests that these autoantigens may be the most important ones in the pathophysiology in AD that are currently known. However, it is important to note that these data are based on only a few studies with relatively small sample sizes.

One study could not confirm a relationship between IgE and IgG subclass reactivity [51], which means that IgE autoantibodies can react to other antigens as IgG autoantibodies, while others found a positive association between IgG and IgE autoantibody reactivity’s [57]. Therefore, the role of this isotype relationship remains unclear. In addition, IgE responses to autoantigens can be boosted by seasonal exposure to pollen allergens in sensitized patients [51]. The presence of both exogenous allergens and autoallergens may therefore accelerate the immune response. It remains to be investigated what comes first; the exogenous allergens that facilitate the development of autoimmunity, or vice versa?

## Pathomechanisms of autoimmunity

### Autoreactive T cells

One of the most critical functions of the immune system is to avoid an effector response against self-antigens. During development in the thymus, T cells undergo positive selection for self-major histocompatibility complex (MHC) reactivity and a negative selection to destroy T cells with a high T cell receptor (TCR) affinity for self-peptides. T cells with lower affinity to self-peptides escape this selection and enter the periphery. The peripheral T cell population contains therefore approximately 4% of self-reactive T cells in healthy individuals [58]. Normally, peripheral tolerance mechanisms, such as suppression by T regulatory cells, anergy and ignorance, prevent these self-reactive T cells to become fully activated and cause pathological damage [59–61]. Like T regulatory cells, self-reactive T cells may even exert beneficial functions and contribute to tissue repair, to maintain tissue homeostasis, and stimulate the immune response against pathogens [58]. When the immune system is unable to distinguish between self and non-self-antigens, autoimmunity can develop as a consequence of loss of self-tolerance [62, 63]. Several pathways may underlie development of autoimmunity: (1) cross-reactivity due to molecular mimicry of exogenous and self-antigens, (2) epitope spreading; an autoimmune response secondary to the release of self-antigens due to tissue damage or chronic inflammation [64], (3) bystander activation; unspecific activation of T and B cells independent of TCR or B cell receptor signaling, such as cytokines, co-receptor expression and gap junctions [63], and (4) downregulation of regulatory T cells. Additional file 1: Table S3 provides an overview of studies on T-cell autoreactivity in AD.

### Molecular mimicry

**Fig. 2:**
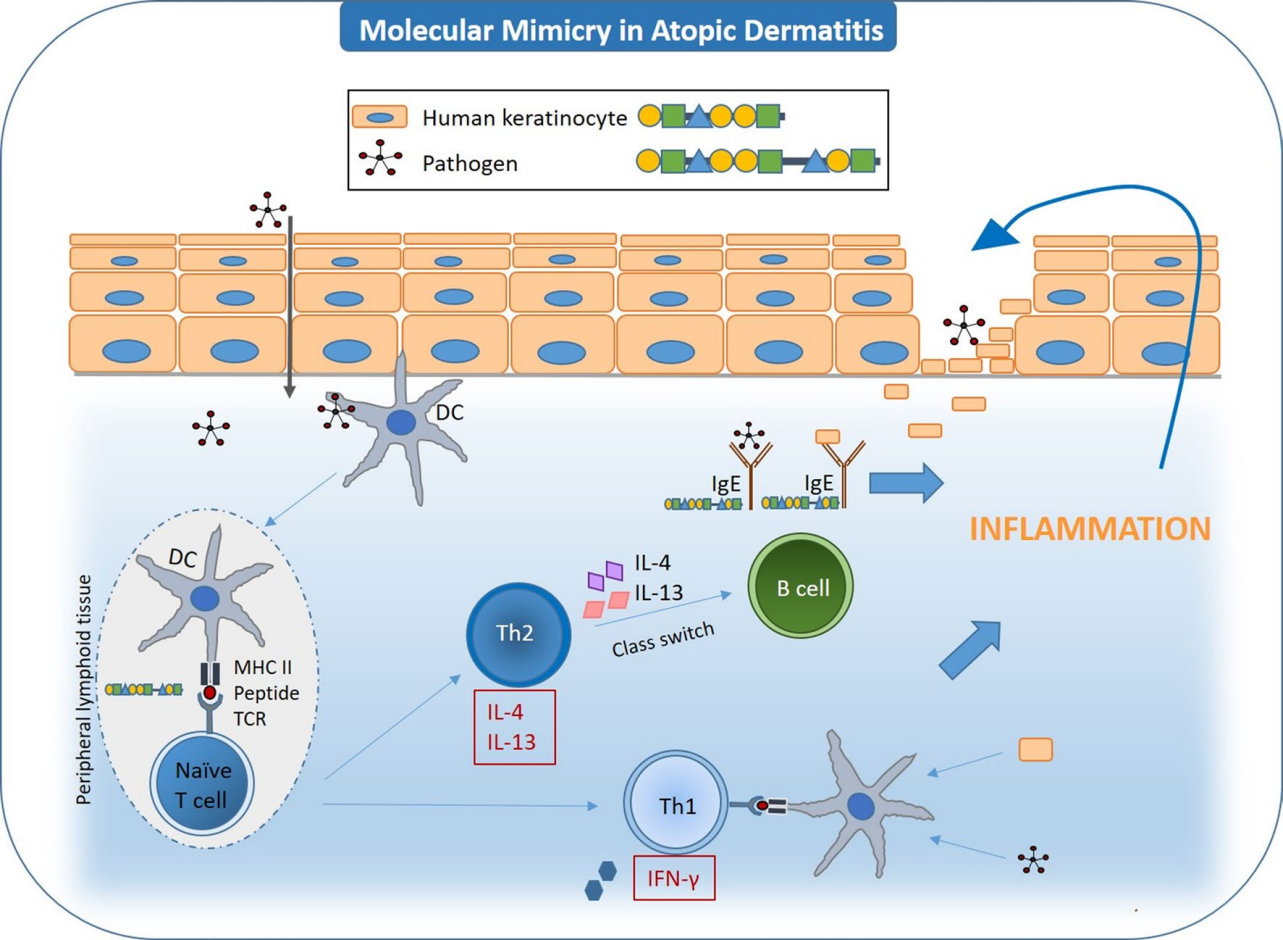
Molecular mimicry can lead to IgE antibody production against self-antigens. An impaired barrier function facilitates access to environmental allergens and pathogens resulting in allergic sensitization and specific IgE antibody production. In the case of structural homologies of the epitopes (molecular mimicry), this can result in cross reactivity between exogenous antigens and self-antigens. *DC* dendritic cell, *Th2* T helper 2 lymphocytes, *IgE* immunoglobulin E, *IL-4* interleukin-4

Molecular mimicry can occur when exogenous antigens activate autoreactive T or B cells due to structural homologies between the epitopes of foreign-antigens and self-antigens leading to autoimmunity [65]. Recognition of a range of MHC complexes is also called polyreactivity of TCRs or cross-reactivity. Evolutionary well conserved peptides in humans can share similar protein structures with pathogens which may lead to molecular mimicry after exposure to the pathogens. Serum IgE from subjects sensitized to thioredoxins (Trx) of *Malassezia sympodialis* (Mala s 13), *Malassezia furfur* or *Aspergillus fumigatus* were shown to cross-react with human Trx proteins leading to immediate allergic responses [22, 33, 66]. Likewise, the stress-inducible enzyme manganese superoxide dismutase (MnSOD) of fungal origin can also induce a humoral response against human MnSOD based on molecular mimicry. It was shown that specific-IgE to human MnSOD correlated with disease activity in 29/67 patients with AD [34]. Thus, molecular mimicry leads to antigen-specific T cells and antibodies that cross-react to both the foreign-peptide and the human-peptide. The latter can be demonstrated in an animal model using human peptides for sensitization subsequently followed by re-exposure to the human peptide leading to a Type-I hypersensitivity reaction. Antigen challenge of mice that were sensitized with human α-NAC showed an immediate allergen response with a mixed Th1 and Th2 reactivity and the presence of IgE and IgG antibodies against murine and human α-NAC [67]. Sequence similarities to human proteins can lead to binding of self-peptides in the skin and T cell activation, which may contribute to the pathophysiology of AD (Fig. 2). However, structural homology is unlikely to be a single pathway to elicit an autoimmune response and other factors such as loss of central tolerance, bystander activation and environmental or genetic factors also play a role in the development of autoimmunity [65].

### Epitope spreading

Tissue damage or an impaired barrier function is crucial in the development of allergic sensitization. Beside genetic mutations, which can facilitate barrier defects, skin damage can be caused by mechanistic stress, such as scratching sequel of itch. In addition, exogenous factors [68–70] or mediators that are released during an ongoing chronic inflammation (IL-4, histamine, mast cell chymase) [71, 72] can facilitate an impaired barrier function. Endogenous proteins that are released following tissue damage can prime self-reactive T and B cells and become specific targets for autoantibodies. The role of epitope spreading is difficult to prove in humans, but is likely to contribute to AD. Tissue debris can be processed and presented on MHC-class II by peripheral antigen presenting cells (APCs) in lymphoid tissues. Activated and differentiated Th1 cells may migrate to the skin where they are re-exposed to the autoantigens by the resident APCs, such as dendritic cells and Langerhans cells. Autoreactive Th1 cells will then release cytokines and chemokines, such as tumour necrosis factor (TNF)-α, interferon (IFN)-γ and lymphotoxin, which directly accelerate further tissue damage. Macrophage inflammatory protein 1α and IFN-γ activate monocytes to differentiate into macrophages and facilitate bystander tissue destruction [64]. Cutaneous lymphocyte-associated antigen (CLA) is a skin-homing receptor expressed by more than 90% of skin-infiltrating T cells and 10–25% of the circulating T cells [73]. CLA^+^CD45RO^+^ T cells recognize allergens related to the skin and release higher levels of IL-13 and some IL-4 or IFN-γ, while CLA^−^ CD45RO^+^ T cells produce higher amounts of IFN-γ [73, 74]. Higher amounts of IL-13 and IL-4 were released from CLA^+^ T cells of patients with AD compared to those of healthy controls [73]. IL-13 and IL-4 are needed for the isotype switch of B cells, while IFN-γ is a key factor that drives the Th1 response. Evidence was found that Hom s4 can induce the production and release of IFN-γ both in non-atopic and atopic individuals [37]. IL-17 was significantly upregulated by the α-chain of the nascent polypeptide-associated complex (α-NAC or Hom s2) [75] (Additional file 1: Table S3). In addition, the CLA^+^CD45RO^+^ T cells of patients with AD produce higher amounts of IL-31 than in healthy individuals [76]. IL-31R is expressed by keratinocytes and infiltrating macrophages [76], but also on sensory neurons [77] resulting in pruritus and inflammation, the hallmarks of AD, which drive the vicious cycle of skin damage.

**Fig. 3:**
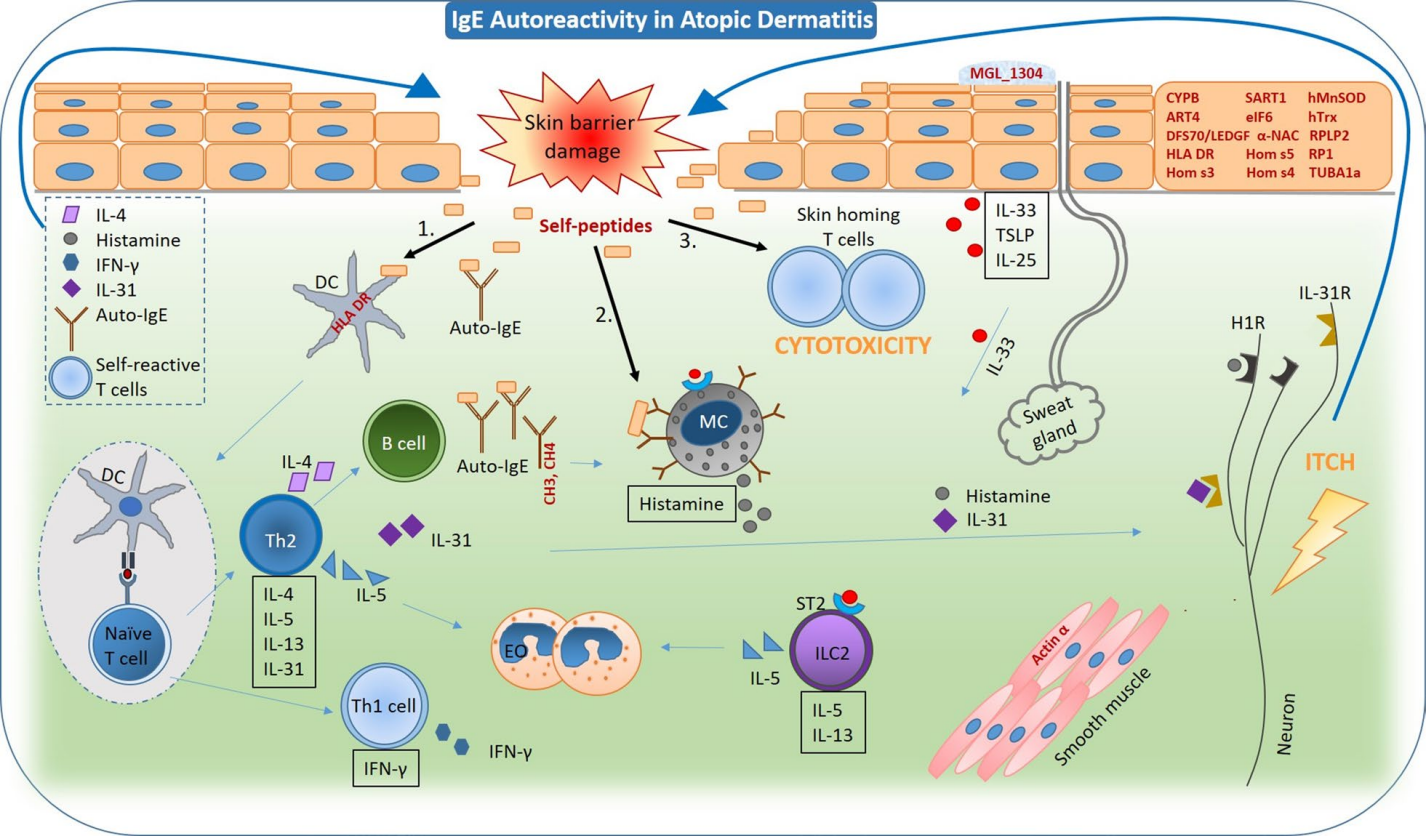
Hypothetic cellular pathways and targets of autoreactivity in atopic dermatitis. Skin damage can lead to release of self-peptides resulting in allergic sensitization to autoantigens via antigen presentation by dendritic cells (DC) to naïve T cells and class switch of B cells. Mast cells sensitized with IgE autoantibodies can directly interfere with the self-peptides that are released following skin damage resulting in histamine release. T cells with low binding affinity to self-peptides may escape selection and depletion in the thymus. Possibly, this population of cells may also be attracted to the skin where they are exposed to the self-peptides of the damaged skin. Cytokines (IL-4 and IFN-γ) produced by T-cells, and histamine by MC can directly exacerbate the skin lesion. Additionally, neurons in the skin can bind histamine and IL-31 specifically to the histamine 1 receptor (H1R) and IL-31 receptor (IL-31R) which results in itch and may lead to the chronicity of the impaired barrier function. *Th2* T helper lymphocyte, *EO* eosinophils, *ILC2* innate lymphoid cell, *IgE* immunoglobulin E, *IL* interleukin, *IFN-γ* Interferon gamma, *TSLP* thymic stromal lymphopoietin

It still remains speculative what the exact contribution of autoreactivity is in the pathophysiology of AD. A summary of the possible pathways and targets that may be involved in the process of allergy-autoimmunity in AD is presented in Fig. 3.

## New treatments as a model for better understanding of atopic dermatitis pathophysiology

Given the multiple pathomechanisms that contribute to AD, it is not always possible to find the underlying factor that drives the disease. Therefore, treatment responses of patients vary greatly. Patients with mild AD are relatively well-controlled with conventional therapies. Besides, in recent years, significant progress has been made for the development of pathway-specific biologic therapies for patients with severe and uncontrolled AD. Variations in treatment responses to pathway-specific effects can help to identify clinically relevant disease endotypes.

Patients with AD often show high serum IgE levels. Treatment with the recombinant humanized monoclonal antibody omalizumab, targeting the Fc of IgE, was thought to be a promising approach to control AD. However, a low response rate to omalizumab was found [78]. A systematic review of omalizumab treatment in severe AD showed that 62.5% had some benefit of the treatment, while no effect was found in 37.5% of the patients [79]. Other skin related diseases, such as bullous pemphigoid [80] and chronic spontaneous urticaria [81] can be successfully treated with omalizumab, highlighting the importance of IgE in the disease pathophysiology. As both bullous pemphigoid and chronic spontaneous urticaria are associated with the presence of IgE autoreactive antibodies, omalizumab could possibly be effective in patients with AD and IgE autoantibodies.

The monoclonal antibody rituximab induces B cell depletion and impairs the interaction between autoreactive Th cells [82] and antigen-presenting B cells. Rituximab also reduces the expression of CD40 and CD80 on B cells as well as the expression of CD40L, CD69, ICOS and HLA-DR on CD4^+^ Th cells, reducing the interaction between immune cells [83]. Rituximab directly induces CD4^+^CD25^+^ regulatory T cells [84]. Rituximab is effective to treat patients with autoimmune diseases, such as SLE, pemphigus vulgaris and rheumatoid arthritis. This highlights the role of B cells and autoreactive T cells in these diseases. Only limited trials have been published on rituximab treatment in AD with some conflicting results. Rituximab treatment can improve AD [85] or can be combined with omalizumab [86]. However, treatment of two patients with AD could not confirm these data [87]. It might be interesting to study the effect of rituximab on clinical improvement on selected patients with AD and autoreactivity.

IL-5 is a key player in the pathogenesis of eosinophilic diseases. Anti‐IL‐5 antibody (mepolizumab) therapy is effective to treat a subgroup of patients with persistent airway eosinophilia and moderate to severe asthma [88] as well as eosinophilic esophagitis [89]. The effect of mepolizumab on clinical improvement has also been studied in patients with AD [90]. A clinical trial including 18 actively and 22 placebo treated subjects demonstrated reduced numbers of blood eosinophils after mepolizumab and significant but only moderate improvement [90]. More evidence is needed to state that IL-5 may not be the crucial cytokine the pathomechanism of AD.

Systemic treatment with cyclosporine was shown to improve skin symptoms in patients with AD, and to reduce IgE autoreactivity [53, 91]. After ceasing treatment with cyclosporine, autoreactivity reappeared. Cyclosporine treatment suppresses the pathways involved in the autoimmune process, such as suppression of T cells as well as the restoration of the skin barrier which reduces the contact to the (auto)antigens [53].

Dysregulation of the Janus kinase/signal transducer and activator of transcription (JAK/STAT) pathway can contribute to autoinflammatory diseases including AD, psoriasis, rheumatoid arthritis, cutaneous lupus erythematosus, lichen planus [92]. Inhibitors of the JAK/STAT pathway are small molecules with immunosuppressive effects targeting JAK/STAT-mediated pathways by preventing the phosphorylation of JAK kinases, and thereby activation of STAT transcription factors. Most pathways in the AD pathogenesis are regulated by JAK/STAT kinases, such as the IL-4 receptor α (binding of IL-4 and IL-13). Therefore, treatment with JAK/STAT inhibitors can result in reduced cytokine production and effectively reduce the immune response. Interestingly, treatment with tofacitinib, a JAK1/JAK3 inhibitor, can also directly target keratinocytes and fibroblasts in 3D human skin equivalents in absence of immune cells [93]. IFN-γ signaling is mediated by the JAK/STAT pathway and is downregulated by JAK2 inhibitors, such as ruxolitinib (JAK1/JAK2 inhibitor). Oral or topical tofacitinib has been shown to be effective to treat autoimmune inflammatory diseases in phase 2 and 3 studies and is a promising treatment in AD [94–100]. It is unknown whether JAK/STAT inhibition may contribute to reduced levels of IgE autoantibodies. If so, it may affect IgE autoreactivity in an indirect way.

Dupilumab is a human monoclonal antibody against the IL-4 receptor α. Phase 3, clinical trials have proven the clinical effectiveness of dupilumab treatment in patients with moderate/severe AD [101–103]. A recent study of 138 adult patients with severe AD also confirmed the clinical effectiveness with improved symptoms of AD and self-reported clinical scores for pain/discomfort, itch, anxiety and depression, and quality of life [104]. The responses to both dupilumab treatment and JAK inhibitors in patients with severe AD confirm the importance of the cytokines IL-4 and IL-13 in the disease pathomechanism. Future studies are needed to evaluate the effects on the production of IgE autoantibodies in patients with AD.

## Conclusion

Increased understanding of the contribution of IgE autoantibodies to the pathophysiology of AD may lead to improved diagnosis, treatment and prognosis. In addition, early detection may increase successful disease management. Therefore, moving from phenotypes to endotypes will help to understand the pathomechanism of AD, prediction of the course of disease and development of personalized therapy. Progress has been made to find evidence for the presence of IgE autoantibodies in AD and many targets have been identified (Table 2). Based on the body of evidence in the literature, it can be accepted that IgE autoantibodies and T cells against epitopes in the human skin can contribute to the pathophysiology of AD. However, the clinical relevance of IgE autoantibodies and self-reactive T cells in AD is still unclear, although, there are many studies showing an association between IgE autoreactivity and severity of clinical symptoms.

Currently, many questions on the role of autoantibodies in the pathomechanism of AD are still open:

- Is there an autoreactive endotype of AD or is this an epiphenomenon?
- Do the IgE autoantibodies start to develop in early childhood?
- Can IgE autoantibodies have a beneficial role in healthy individuals especially in children?
- Can this role show a switch to a pathogenic response?
- Do they expand prior to disease progression and can they be used as predictive marker?
- What is the subtype of self-reactive T cells and what is their exact role in AD?
- What are the therapeutic implications of the presence of IgE autoreactivity in patients with severe AD?

Recently, four distinct clusters of patients with AD have been identified based on serum biomarker- and cytokine profiles regardless their clinical presentation [105]. Identification of biomarkers can identify downstream pathways that are involved in AD and clustering based on serum biomarker profiles may therefore be useful to explain the biological mechanisms of AD. It is clear that more research is warranted to find answers to the questions. Future perspectives include the clinical characterization of the individuals with AD and autoreactivity, the onset of IgE autoantibodies development, investigation of the contribution of the IgE autoantibodies and self-reactive T cells to the severity and chronicity of AD as well as the response to immune regulatory treatments in this group of patients.

## Supplementary information

**Additional file 1: Table S1.** Systematic search strategy. **Table S2.** Systematic search on IgE autoantibodies in patients with atopic dermatitis. AD: atopic dermatitis, RC: rhino conjunctivitis, NA: nonatopic, HC: healthy controls without allergic symptoms, PS: psoriasis, CA: contact allergy, UA: urticaria. **Table S3.** Autoreactive T cells in patients with atopic dermatitis. AD: atopic dermatitis, RC: rhinoconjunctivitis, HC: healthy controls, NA: non-atopic, CD: chronic dermatoses, PS: psoriasis, ns: non-sensitized, ABPA: allergic bronchopulmonary aspergillosis. * median.

## Data Availability

We performed a systematic search on Pubmed.

## Abbreviations

AD: Atopic dermatitis
ANA: Antinuclear antibodies
APC: Antigen presenting cell
CLA: Cutaneous lymphocyte-associated antigen
DFS: Dense fine speckles
hTrx: Human thioredoxins
hMnSOD: Human manganese superoxide dismutase
IFN-γ: Interferon gamma
Ig: Immunoglobulin
IL: Interleukin
JAK: Janus kinase
MHC: Major histocompatibility complex
STAT: Signal transducer and activator of transcription
TCR: T cell receptor
TNF-α: Tumor necrosis factor alpha
Th1: T helper 1 lymphocytes
Th2: T helper 2 lymphocytes
