# Supplementary material for: IgE autoantibodies and autoreactive T cells and their role in children and adults with atopic dermatitis
Source: Clin Transl Allergy. 2020 Aug 3;10:34. doi: 10.1186/s13601-020-00338-7 (PMC7398196; doi:10.1186/s13601-020-00338-7)
Supplement: Supplementary file 1 — Additional file 1: Table S1. Systematic search strategy. Table S2. Systematic search on IgE autoantibodies in patients with atopic dermatitis. AD: atopic dermatitis, RC: rhino conjunctivitis, NA: nonatopic, HC: healthy controls without allergic symptoms, PS: psoriasis, CA: contact allergy, UA: urticaria. Table S3. Autoreactive T cells in patients with atopic dermatitis. AD: atopic dermatitis, RC: rhinoconjunctivitis, HC: healthy controls, NA: non-atopic, CD: chronic dermatoses, PS: psoriasis, ns: non-sensitized, ABPA: allergic bronchopulmonary aspergillosis. * median. [file 13601_2020_338_MOESM1_ESM.docx]

**Table S1: Systematic search strategy**

| Search | PubMed Search Query 03 October 2019 | Hits |
| --- | --- | --- |
| #1 | Atopic AND Dermatitis | 27232 |
| #2 | atopic AND eczema | 7162 |
| #3 | autoreactivity OR autoreactive OR autoreactiv* OR self-reactiv* | 11389 |
| #4 | anti-IgE OR (anti AND IgE) OR (auto AND IgE) OR (auto AND anti AND IgE) | 12233 |
| #5 | autoantigen* OR autoallergen | 26699 |
| #6 | autoallergy | 71 |
| #7 | autoimmunity OR autoimmune OR (autoimmune AND disease*) | 197058 |
| #8 | autoantibodies OR (IgE AND autoantibodies) OR (IgG AND autoantibodies) | 11087 |
| #9 | autoreactive AND T cells | 5941 |
| #10 | #1 OR #2 | 28190 |
| #11 | #3 OR #4 OR #5 OR #6 OR #7 OR #8 OR #9 | 291205 |
| #11 | #10 AND #11 | 1552 |

**Table S2: Systematic search on IgE autoantibodies in patients with atopic dermatitis**

| **References** | **Study design** | **Population size (n)** | **Age: mean (**$\pm$**sd/range)** | **AD diagnostic criteria** | **Measurement of autoreactivity** | **Prevalence of auto-IgE in patients with AD** | **Prevalence of auto-IgE in control group** |
| --- | --- | --- | --- | --- | --- | --- | --- |
| Aichberger et al., 2005 (37) | Case-control | AD, n=12  RC without AD, n=11  NA, n=6  NA with CD, n=3  NA with PS, n=3 | 38.5 (20-72) | Hanfin and Rajka | Western Blot  Serum IgE against epithelial cell line A 431 and rHom s 4 | A342: 91,7% (11/12)  Hom s 4: 16,7% (2/12) | 0% (0/23) |
| Altrichter et al., 2008 (35) | Case-control | AD, n=192  HC, n=5 | AD: 39 (18-80)  NA: 3 (25-64) | Hanfin and Rajka | Western Blot  Serum IgE against epithelial cell line A 431 and/ or epidermis | 28% (54/192) | 0% (0/26) |
| Guarneri et al., 2015 (109) | Case-control | AD, n=27  HC, n= 27 | AD: 22.2(12.2)  Range: 5-49 | Hanfin and Rajka | Skin prick test against hMnSOD | 14.8% (4/27) | 0% (0/27) |
| Hide et al., 2002 (54) | Case-control | AD, n=66  AR without AD, n=7  HC, n=27 | AD: 24.7 (5.2)  AR without AD: 24.6 (4.8)  HC: 27.8(6.7) | Rajka and Langeland | Skin test against autologous sweat | 84.8% (56/66) | HC: 11.1% (3/27)  AR: 71.4% (5/7) |
| Kawamoto et al., 2003 (30) | Case-control | AD, n=40  HC, n= 41 | AD: 26.4 (12-44)  HC: 29.2 (19-54) |  | ELISA  CTL-directed peptides  SART3_109_  SART3_315_  CypB_84_  CypB_91_  ART4_75_ | 2.5% (1/40)  37.5% (15/40)  15% (6/40)  27.5% (11/40)  17.5% (7/40)  10% (4/40) | 4.9% (2/41)  29.3% (12/41)  17.1% (7/41)  29.3% (12/41)  31.7% (13/41)  12.2% (5/41) |
| Kortekangas-Savolainen et al., 2004 (33) | Case-control | IgE- mediated AEDS: n=27  HC: n=6  UA: n=4  PS: n=3 | AD: 33(11)  HC: 47 |  | Western blot  Serum IgE against keratinocytes | 37% (10/27) | 0% (0/13) |
| Mitterman et al., 2008 (38) | Case-control | AD: n=11  RC: n=7  CA: n= 5  NA: n=9 | AD: 31.9(14.9)  Controls: 35.2(10.9) | Hanfin and Rajka | Immunoblotting  IgE against epithelial cell-line A431 | 72.7% (8/11) | 0% (0/12) |
| Mothes et al., 2005 (20) | Case-control | AD: n=174  PS: n=10  HC: n=26 | AD: 35.4 (14.8)  HC: 36.5 (16.5) | Hanfin and Rajka | Immunoblotting  IgE against epithelial cell-derived antigens | 23% (40/174) | 0%(0/10) |
| Natter et al., 1998 (19) | Case series | AD, n=51 | AD: 26.9 (18.6)  Range: 1-63 | Hanfin and Rajka | Western blot  Serum IgE against endothelial cells and A431 | 43.1% (22/51) | / |
| Ochs et al., 2000 (57) | Case series | AD: n=64 | AD: 24.4(4-34) | Hanfin and Rajka | Western blot  IgE against DFS70 | 62.5% (10/16) | 0% (0/1) |
| Schmid-Grendelmeier et al., 2005 (34) | Case-control study | AD, n=69  HC, n=5  PS, n=13  ABPA, n=11  *A.fumigatus*, n=13 | AD: 29.3(5.6)  Other: 25.3 | Hanfin and Rajka | ELISA  against rhMnSOD | 42% (29/69) | ABPA: 100% (11/11)  Other: 0% |
| Tanaka et al., 2006 (55) | Case-control | AD, n=62  PS, n=13  HC, n=46 | AD: 24.0 (7.5)  Range: 2-43  PS: 53.4 (17)  Range: 29-73  HC: 28.4 (9)  Range: 1-52 | / | Dot blotting of IgE against purified sweat antigen | 77.0% (47/61) | 8.7% (4/46) |
| Valenta et al., 1996 (17) | Case-control | AD, n=20  HC, n=28 | AD: 35.3(12.3)  HC: 33(11.4) | Hanifin and Rajka | Western Blot  IgE against endothelial cells, platelets, fibroblast and epithelial cells | 60% (12/20) | 0% (0/28) |
| Watanabe et al., 2011 (31) | Case-control | AD, n=61  HC, n=20 | AD: 26.2 (9.8) Range: 13-59  HC: 33.1 (9.5) Range: 22-57 | Hanifin and Rajka | ELISA against DFS70 | 14.8% (9/61) | 0% (0/20) |
| Zeller et al., 2009 (36) | Case-control study | AD, n=71  PS, n=12  HC, n=24 | AD: 33.35(12.7)  HC: 29(3.71) | Hanifin and Rajka/EAACI | Immunoblotting and ELISA  actin-alpha, eIF6, RPI, HLA-DR-alpha and tubulin-alpha | AD: 71,8% (51/71) | Non-AD: 72,2%(13/18) |

**Table S3: Autoreactive T-cells in patients with atopic dermatitis**

| References | Population size (n) | Age: mean ($\pm$sd/range) | Diagnostic criteria for AD | Stimulation with (auto)antigen | T cell autoreactivity in patients with AD |
| --- | --- | --- | --- | --- | --- |
| *Aichberger et al., 2005* (37) | AD, n=12  RC no AD, n=11,  NA, n=6,  CD, n=3,  PS, n=3 | AD: 38.4 (20-72) | Hanifin and Rajka | Hom s 4 | Th1-biased immune response through IFN-ɣ |
| *Balaji et al., 2011* (114) | AD s, n=3, AD ns, n=3  PS ns, n=3,  HC, n=5 | AD: 42 (21-66) | Hanifin and Rajka | Mala s 13/hTrx | Increased proliferation of lymphocytes in PBMCs. Mala s 13-specific TCL and TCC cells show full cross-reactivity with hTrx. Mala S 13-specific TCC are mainly CD4^+^ and express CLA. TCCs belonging to T_H_1, T_H_2, T_H_17 and T_H_22 phenotypes |
| *Heratizadeh et al., 2011* (115) | AD, n= 30  HC, n= 1 | AD: 36 (12) | Hanifin and Rajka | α-NAC | Proliferation of CCR4^+^ and CLA^+^ |
| *Hradetzky et al., 2014* (75) | AD, n=11, AD ns, n=12  HC, n=18 | AD: 40 | SCORAD | α-NAC | Increased secretion of IFN-ɣ, IL-17 and IL-22 in α-NAC stimulated PMBCs. Lower levels of IL-10 produced in α-NAC stimulated PMBC’s |
| *Kapitein et al., 2013* (116) | AD, n= 55  HC, n=30 | AD: 8.8 (1.5-17.5)  HC: 8.6 (1.2-17.3) | Hanifin and Rajka | hsp60 | Increased presence of hsp60 in AD children  Higher T cell proliferation  High levels of IFN-ɣ and less IL-10 production by hsp60-reactive T cells (CD4^+^CD14^+^)  hsp60-induced CD4^+^CD25^+^CD127^-^ T cells are not suppressive in vitro |
| *Lind et al., 2009* (117) | AD, n=78  HC, n=45 | AD: 29 (18-65)  HC: 39 (20-64)* | UK Working Party’s Diagnostic criteria | *M. sympodialis* | Elevated plasma levels of IL-18  Activation of iNKT-cells, release of IFN-ɣ |
| *Roesner et al., 2016* (106) | AD, n=12  HC, n=9  HLA-A*02^+^ AD, n=15  HLA-A*02 PS, n=3  HLA-A*02^+^ HC, n= 12 |  | Hanifin and Rajka | α-NAC | Strong proliferation of α-NAC specific CD8^+^ T cells, secreting IL-4 and IFN-ɣ. Higher frequencies of α-NAC specific CD8^+^ T cells vs HC. Increased numbers of CD8^+^/CD45RA^-^/CD127^-^T_EM_ and CD8^+^/CD45RA+/CD27^-^ T_EMRA_ CD8^+^ subsets |
| *Schmid-Grendelmeier et al., 2005* (34) | AD, n= 69  PS, n= 13  *A. fumigatus*, n=13  ABPA, n=11; HC, n=5 | AD: 29.3 (5.6)  HC: 22.3 (4.9) | Hanifin and Rajka | hMnSOD  *M. sympodialis*  *A. Fumigatus*  Asp f6 | Higher proliferation rate of T cells in hMnSOD sensitized patients |
